# Membrane Filtration-Assisted Enzymatic Hydrolysis Affects the Biological Activity of Potato Juice

**DOI:** 10.3390/molecules26040852

**Published:** 2021-02-06

**Authors:** Przemysław Łukasz Kowalczewski, Anna Olejnik, Iga Rybicka, Magdalena Zielińska-Dawidziak, Wojciech Białas, Grażyna Lewandowicz

**Affiliations:** 1 Department of Food Technology of Plant Origin, Faculty of Food Science and Nutrition, Poznań University of Life Sciences, 31 Wojska Polskiego St., 60-624 Poznań, Poland; 2 Department of Biotechnology and Food Microbiology, Poznań University of Life Sciences, 48 Wojska Polskiego St., 60-627 Poznań, Poland; anna.olejnik@up.poznan.pl (A.O.); wojciech.bialas@up.poznan.pl (W.B.); grazyna.lewandowicz@up.poznan.pl (G.L.); 3 Department of Technology and Instrumental Analysis, Poznań University of Economics and Business, Al. Niepodległości 10, 61-875 Poznań, Poland; iga.rybicka@ue.poznan.pl; 4 Department of Biochemistry and Food Analysis, Faculty of Food Science and Nutrition, 48 Mazowiecka St., Poznań University of Life Sciences, 60-623 Poznań, Poland; magdalena.zielinska-dawidziak@up.poznan.pl

**Keywords:** antiproliferative activity, antioxidant activity, cancer cells, cytotoxicity, in vitro study, nutritional value

## Abstract

The results of recently published studies indicate that potato juice is characterized by interesting biological activity that can be particularly useful in the case of gastrointestinal symptoms. Moreover, the studies also described the high nutritional value of its proteins. This article is a report on the impact of the enzymatic hydrolysis of proteins combined with membrane filtration. The obtained potato juice protein hydrolysate (PJPH) and its concentrate (cPJPH) were characterized in terms of their nutritional value and biological activity. The amino acid profile and scoring, the content of mineral compounds, and the antioxidant and in vitro cytotoxic activity were assessed. The study proved that the antioxidant activity of PJPH is higher than that of fresh potato juice, and the cytotoxicity against human gastric carcinoma cell line (Hs 746T), human colon cancer cell line (Caco-2), human colorectal adenocarcinoma cell line (HT-29), and human normal colon mucosa cell line (CCD 841 CoN) showed biological activity specifically targeted against cancer cells. Therefore, it can be concluded that the membrane filtration-assisted enzymatic hydrolysis of potato juice proteins may increase their biological activity and allow for potato juice to be used in the production of medicinal preparations.

## 1. Introduction

In recent years, problems with the management and appropriate use of by-products in the food industry have become an increasing challenge. One of the most interesting side streams in the food industry is potato juice (PJ), arising during the production of potato starch [1]. According to the published data, up to 500 kg of PJ can be made from 1 ton of potatoes [2,3]. PJ consists of both mineral compounds and organic substances, primarily proteins. Non-protein organic substances mainly include vitamins (B1, B2, B6, PP, C, and E), as well as antinutritional substances (phytates) and even toxic substances (glycoalkaloids) [4,5,6,7]. Potatoes reveal huge intraspecific diversity, nevertheless, it should be noted that the most popular varieties of potatoes are an extraordinarily rich source of macro- and microelements. The iron content in 100 g of the dry matter of juice from the most popular potato varieties is over ten times the recommended daily intake (RDI) for this element. Potassium and calcium are present at 400% and 150% of the recommended daily values, respectively [8]. The difference in the content of toxic alkaloids in individual potato varieties varies within five orders of magnitude; however, the content of glycoalkaloids (GAs) in potatoes intended for consumption is much lower than the permissible limits [8,9]. Currently, PJ is used as a feed component, a limited use which fails to recognize and exploit the full potential of this material. Some researchers attempted to use PJ as an ingredient in microbial media and to obtain valuable metabolites [10,11,12,13,14]. However, it seems more promising to use it for the production of health-promoting foods [15,16,17], as PJ stands out not only for its high nutritional value, but also for its distinctive biological activity.

Freshly squeezed PJ was used in European German-speaking countries as a medicine in traditional folk medicine. It was believed that it is effective in treating stomach ulcers. At the end of the 19th century, Swiss physician Maximilian Bircher-Benner initiated the use of PJ as a therapeutic agent. However, the scientific verification of the effectiveness and safety of this material as a medicine only began in the 21st century [18,19]. The first studies indicated the key role of the protein fraction in the therapeutic (mainly anti-inflammatory) effects of PJ. The protease inhibitor fraction was found to be particularly active [20,21]. Later studies demonstrated the broader biological activity of PJ that was not always associated with the protein fraction [5,6,22]. The cytotoxic activity of PJ towards intestinal cancer cells is particularly worthy of attention [22,23]. The individual substance responsible for this activity has not yet been identified, although the key role of GAs has not been excluded. At the end of the 20th century, it was shown that GAs reveal in vitro activity against neoplastic cells [24]. Subsequently, it was proven that solanine and chaconine have the ability to induce tumor cell apoptosis [25,26]. It should be emphasized that biological activity, including anti-inflammatory activity, did not decline as an effect of thermal treatment [22,27]. Moreover, the anti-inflammatory activity of PJ subjected to thermal treatment was demonstrated in in vivo studies. In particular, Kujawska et al. [28] showed that spray-dried PJ could be used for ameliorating inflammation-related diseases of the gastrointestinal tract.

The enzymatic hydrolysis of proteins has received increasing interest over the last year because this process makes it possible to achieve multiple, non-contradictory purposes. Primarily, it makes it possible to extract protein fractions from unconventional sources and make them more digestible [29]. Moreover, enzymatic hydrolysis enables the reduction of the allergenicity of nutritionally important proteins [30]. Most often, however, the possibility of producing bioactive peptides is exploited [31,32,33,34]. This process was extensively studied for whey proteins and can be especially efficient when it is performed using a membrane reactor [35,36,37,38]. Numerous studies regarding potato proteins indicated the potential of enzymatic hydrolysis for obtaining bioactive products; however, the possibility of using a membrane reactor for that process has not yet been studied [39,40,41,42]. Nevertheless, the use of a membrane reactor, which makes the precise separation of individual fractions possible, may be of key importance for the functional properties of the obtained hydrolysate [43]. In our previous work, we showed that different preparations derived from PJ could be used for the manufacturing of functional foods (pasta, frankfurters, breads, or pâtés); however, the attractiveness of the products to consumers strongly depends on the form of protein in the foods [15,16,17,44]. Moreover, the method used for the isolation of the protein fraction from PJ also influenced the biological activity of the products [22,23,28]. The application of a membrane reactor for the enzymatic hydrolysis of the protein fraction of PJ could result in a product with high nutritional value, attractive functional properties, and increased biological activity. Therefore, the aim of the study was to verify the hypothesis presented above.

## 2. Results and Discussion

### 2.1. Chemical Composition and Nutritional Value of Potato Juice Protein Hydrolysate

Table 1 presents the results of the protein and mineral compound content in the analyzed fresh potato juice (PJ), the potato juice protein hydrolysate (PJPH), and the concentrated potato juice protein (cPJPH). The use of an ultrafiltration module in the enzymatic hydrolysis process made it possible to obtain a product containing protein in a soluble form, at a concentration seven times higher than in the raw material. The additional use of a nanofiltration module resulted in a further concentration of the protein, with almost double the quantity. The mineral compounds present in the fresh juice were concentrated more than twenty times, mainly using the ultrafiltration module. If we compare the effect of the membrane-assisted enzymatic hydrolysis with the membrane separation of potato proteins described in our previous work [23], the ratio of the protein to mineral fraction content is different. This phenomenon is mainly related to the use of ultrafiltration modules with different cut-offs. The use of a 5 kDa cut-off ultrafiltration membrane to concentrate the fresh PJ, containing non-degraded protein macromolecules, resulted in a more effective separation of both fractions. The membrane-assisted enzymatic hydrolysis using an ultrafiltration module with a cut-off of 1 kDa and a nanofiltration module with a cut-off of 300–500 Da maintained the valuable minerals in the hydrolysis product.

The observations presented above also reflect changes in the individual ion content (Table 2). Both the PJPH and the cPJPH had a very high and comparable content of K (18.8 g and 19.6 g/100 g, respectively). They also had a low content of Na, at 160 mg/100 g in the PJPH and 176 mg/100 g in the cPJPH, which is nutritionally relevant due to the excessive sodium intake in most of the population worldwide [45,46]. Moreover, the PJPH and the cPJPH had a high content of Mg, Mn, and Cu, but their contents significantly differed between the samples analyzed. The content of Mg in the PJPH was 513 mg/100 g, which corresponded to about 140% of the Nutrient Reference Value (NRV) for this mineral, while the content of Mn was 6.18 mg/100 g (above 300% of the NRV), and the content of Cu was 1.19 mg/100 g (about 120% of the NRV). The cPJPH contained about 180% of the NRV for Mg, 370% of the NRV for Mn, and 200% of the NRV for Cu from a 100 g sample. The content of Ca and Zn was high but did not exceed 100% of the NRV in either the PJPH or the cPJPH. The content of Ca was 20% (PJPH) and 29% (cPJPH) of the NRV, and the content of Zn was 60% (PJHP) and 74% (cPJPH) of the NRV. Only the content of Fe was found to be at the low level of 0.44 mg/100 g and 0.56 mg/100 g for both the PJPJ and the cPJPH, respectively, corresponding to less than 5% of the NRV for this mineral. Heavy metals were mainly concentrated at the ultrafiltration stage, which is related to the effective retention of other ions on the nanofiltration membrane. Specifically, the lead content in the cPJPH was below the limit for food supplements [47].

Potato proteins have a high nutritional value due to their amino acid composition. Essential amino acids (EEA) accounted for 32% of the total amino acids (TAA) in the preparation analyzed in this study. These results were not much lower than the results presented by Gorissen et al. [48], which suggested that potato proteins meet the requirement for EAA, which constituted 38% of the TAA in the potato proteins studied. The PJPH unexpectedly had a lower amino acid score (AAS) than usually observed for potato proteins [23]. As shown in Table 3, the limiting amino acid was leucine, with a content that was merely 3.4% of the TAA (compared to 8.3% suggested by Gorissen et al. [48]). A decreased content of lysine was also noted (3.2% of the TAA). However, in the literature, it was found that the product of lysine degradation could be glutamate [49], and a high content of glutamate and glutamic acid was noted (10.1 g/16 g N) in the PJPH. The amino acid profile could also be influenced by the applied hydrolytic enzymes, the storage conditions [50], and the potato variety used to prepare the PJPH [51]. The PJPH was found to be an excellent source of tryptophan (~167% compared to the WHO/FAO standard [52]).

### 2.2. Antioxidant Activity

The number of factors that pose a direct threat to human health are rapidly increasing as a result of development, industrialization, and urbanization. One such factor is oxidative stress, i.e., the excessive accumulation of free radicals in our bodies, which results in oxidative damage to the cells as well as damage to the DNA, lipids, and proteins, which can lead to a number of diseases, including neurodegenerative diseases [53,54,55,56]. Therefore, there is a constant search for antioxidant compounds, of which plants are precious sources [57,58,59,60]. The content of antioxidant compounds may increase significantly depending on the growth conditions and the action of stress factors [61,62]. A comparison of the published data on the antioxidant activity of fresh potato juice [22] and the hydrolysate obtained in this study (Table 4) showed that the enzymatic hydrolysis of the juice caused a significant, 10-fold increase in antioxidant activity. Moreover, the content of polyphenolic compounds in the analyzed hydrolysate was high, several times higher than the content in potatoes with colored flesh [63], and rich in anthocyanins and polyphenols. Data from the literature confirmed that enzymatic hydrolysis can significantly increase antioxidant activity, as well as release bound polyphenolic compounds [64,65,66].

### 2.3. In Vitro Cytotoxicity Assay

The most interesting, least described, and perhaps most important activity of PJ is probably its cytotoxicity against cancer cells [22,23]. On the basis of the obtained results presented in Table 5, it was found that the hydrolysate of PJ protein has a cytotoxic effect, which can be increased with concentration using membrane separation. Moreover, the use of a nanofiltration membrane to concentrate the obtained hydrolysate additionally increased the biological activity of cPJPH. By comparing the IC_50_ values, it was found that the concentration process caused a 1.4–3.3-fold increase in the cytotoxicity of PJPH depending on the cell line tested. The highest cytotoxicity of cPJPH was found in the Caco-2 colon cancer cells. In contrast, the weakest cytotoxic effects were observed in the culture of normal colon mucosa CCD 841 CoN cells. The first cytotoxic dose (IC_10_) of cPJPH to Caco-2 cells was 7.3-fold lower than the IC_10_ to CCD 841 CoN cells. Moreover, the half-maximal inhibitory concentration (IC_50_) and the lethal concentration (IC_90_) of cPJPH determined for Caco-2 cells were significantly lower (3.4- and 1.6-fold, respectively) than those obtained for CCD 841 CoN cells. The high cytotoxic activity of both PJPH and cPJPH was also observed in the colon cancer HT-29 cell culture. As a result of PJPH concentration, a significant increase in cytotoxic potential was found in the stomach cancer Hs746T cell cultures (Table 5). Commonly used anti-cancer drugs cause significant damage to the body of patients because of their non-selective action [67,68,69]. Therefore, there is a need for substances that will act in a more targeted manner. The results of the cytotoxic activity of cPJPH indicated significantly lower IC_50_ doses for neoplastic cells compared to normal cells, which may be of particular interest in the context of further research into the use of PJ ingredients for the treatment of gastrointestinal cancer.

The comparison of the various methods used to process PJ suggests some conclusions regarding the question of which substances in PJ are actually responsible for its cytotoxic effect on cancer cells. While concentration by ultrafiltration using a 5 kDa cut-off membrane resulted in a product with a high content of nutritious protein [23], the membrane-assisted enzymatic hydrolysis presented in this study resulted in a product with significantly increased cytotoxic activity against cancer cells. Moreover, the thermal deproteination of PJ also resulted in products with higher cytotoxic activity against cancer cells compared to the raw materials [22]. These results prove that the anti-proliferative effect of PJ on cancer cells is not related to protein fraction but to other molecules of rather low molecular mass. Surely, this hypothesis requires confirmation with further analyses, but the phenomena observed so far provide important evidence that may direct further studies.

## 3. Materials and Methods

### 3.1. Enzymatic Hydrolysis of Potato Juice Proteins

**Figure 1:**
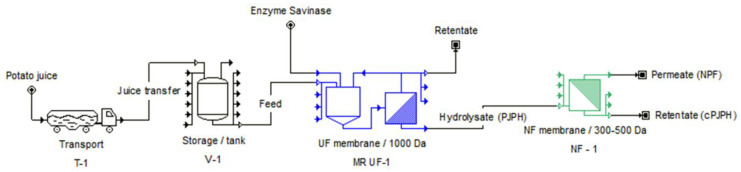
Schematic diagram of the applied membrane separation system for ultrafiltration.

The experimental material, potato juice (PJ), was collected during the starch production season from the production line of “Trzemeszno” Sp. z o.o. Potato Industry Company (Trzemeszno, Poland). The enzyme Savinase^®^ (Sigma-Aldrich, Saint Louis, MO, USA), isolated from the *Bacillus* species, was used as the proteolytic preparation. On the basis of previous preliminary studies (data not shown), an enzyme dose of 4 μL/g of potato protein was adopted. The enzyme was added at the start of the hydrolysis process, according to the initial volume of PJ used in the experiment, and further portions were added every 60 min because of the continuous process, in which the finished hydrolysis product was removed and a new portion of PJ was added in its place. A polyethersulfone spiral-wound ultrafiltration membrane with a molecular weight of 1 kDa cut-off and an area of 3.5 m^2^ (type 3838, SUEZ Water Technologies & Solutions, Budapest, Hungary) was used to perform the enzymatic hydrolysis and, consequently, to obtain a PJ protein hydrolysate (PJPH). The non-hydrolyzed PJ was returned to the initial tank of the system (recirculation). The PJPH was then concentrated on a polyamide thin film composite nanofiltration membrane, with a molecular weight of 300–500 Da cut-off and an area of 4.0 m^2^ (type 3838, SUEZ Water Technologies & Solutions, Budapest, Hungary), to obtain a concentrated fraction of hydrolyzed, soluble potato proteins (retentate, denoted in the text as cPJPH) and a non-protein low molecular weight fraction (filtrate). A flowchart of the process used for these products is presented in Figure 1.

### 3.2. Chemical Analysis

The Kjeldahl method was used to determine the total nitrogen content, which was then used to calculated the protein content using a nitrogen-to-protein conversion factor of 6.25 [70]. The international standard method ISO 763 [71] was used to measure the total ash content.

The concentrations of minerals Ca, Cu, Fe, K, Mg, Mn, Na, and Zn were determined using flame atomic absorption spectroscopy (FAAS) (SpectrAA-800, Varian, Palo Alto, CA, USA) that was preceded by microwave mineralization with nitric acid [72]. The recommendations for Ca, Cu, Fe, Mg, Mn, and Zn were established at the level of the Nutrient Reference Value (NRV) [73]. The contents of the minerals were expressed in g/100 g of the dry mass of the sample.

### 3.3. Amino Acid Composition and Scoring

Histidine (His), isoleucine (Ile), leucine (Leu), lysine (Lys), methionine (Met), phenylalanine (Phe), threonine (Thr), valine (Val), cysteine (Cys), tyrosine (Tyr), glycine (Gly), arginine (Arg), proline (Pro), aspartic acid (Asp), glutamic acid (Glu), alanine (Ala), and serine (Ser) were determined using ultra-performance liquid chromatography (Shimadzu Nexera 2.0, Kyoto, Japan), equipped with a PDA (at 260 nm, sampling rate of 20 points/s) and FL detector (Kyoto, Japan), that was preceded by acidic hydrolysis (110 °C, 23 h) [74]; meanwhile, sulfuric amino acids were prepared by oxidation (4 °C, 16 h) followed by acidic hydrolysis (110 °C, 2.5 h) [75]. The results were expressed in g/16 g N (which is equivalent to g/100 g of protein).

The FAO recommended method was used to calculate the amino acid score (AAS) for adults [52]:

$$\mathrm{AAS}=\frac{\mathrm{essential}\;\mathrm{amino}\;\mathrm{acids}\;\mathrm{contents}\;\mathrm{in}\;\mathrm{PJPC}\;\left[\%\right]}{\mathrm{recommended}\;\mathrm{essential}\;\mathrm{amino}\;\mathrm{acids}\;\left[\%\right]}.$$

### 3.4. Total Phenolic and Antioxidant Activity of the Hydrolysates

The extraction of bioactive compounds was performed using lyophilized products. A 0.5 g sample was extracted with 40 mL of 80% ethanol for 2 h and then centrifuged (4000*× g*, 10 min) using a laboratory centrifuge (Rotofix 32 A, Hettich, Germany). The obtained supernatants were then decanted and filtered through a 0.22 µm filter. The samples were stored in a −20 °C freezer until use.

The Folin–Ciocalteu colorimetric method [76] was applied to measure the total phenolic compounds (TPC) using a spectrophotometer (Multiskan GO, Thermo Fisher Scientific, Vantaa, Finland). The results were expressed as a gallic acid equivalent (mg GAE/g).

The ABTS radical cation decolorization assay was determined by the method of Re et al. [77], with slight modifications that are described elsewhere [62]. A 2 mL sample of the ABTS solution was mixed with 0.98 mL of PBS and 0.02 mL of the PJPH extract. After 6 min of incubation at 30 °C, an absorbance at 734 nm was measured spectrophotometrically (Multiskan GO, Thermo Fisher Scientific, Vantaa, Finland). Trolox was used as a standard, and the results were presented as Trolox equivalents (mg/g of sample).

### 3.5. In Vitro Cytotoxicity Assay

The human colorectal adenocarcinoma cell line HT-29 (Cat. no: 85061109), human gastric carcinoma Hs 746T cell line (ATCC^®^ HTB-135™), human colon cancer Caco-2 cell line (ATCC^®^ HTB-37™), and human normal colon mucosa CCD 841 CoN cell line (ATCC^®^ CRL-1790™) were used in this study and cultured according to the method that was previously described in detail in this report [23]. The cell viability and metabolic activity were determined using the 3-(4,5-dimethylthiazol-2-yl)-2,5-diphenyltetrazolium bromide (MTT) colorimetric assay [78]. The first cytotoxic dose (IC_10_), the median effective concentration (IC_50_), and the lethal dose (IC_90_) were calculated on the basis of the MTT results.

### 3.6. Statistical Analysis

Statistica 13 software (Dell Software Inc., Round Rock, TX, USA) was used to perform a one-way analysis of variance (ANOVA). A post-hoc Tukey HSD multiple comparison test was used to identify statistically homogeneous subsets at α = 0.05.

## 4. Conclusions

The enzymatic hydrolysis of potato juice in a membrane reactor influenced both the nutritional value as well as the biological activity of this raw material. The use of ultrafiltration systems resulted in a product containing soluble proteins in a concentration several times higher than that of a simple enzymatic hydrolysis. Mineral compounds were also concentrated in this process. The additional use of a nanofiltration module resulted in a further concentration of the solutes. The products of the enzymatic hydrolysis of potato juice were characterized by a significantly higher antioxidant activity and concentration of polyphenolic compounds than the raw materials.

It was also found that the enzymatic hydrolysis of potato juice in the reactor with the ultrafiltration membrane separation system increased the cytotoxic activity of the processed material. IC_50_ toxic doses of the hydrolysate for cancer cells were significantly lower than those of fresh potato juice. Moreover, IC_50_ toxic doses of the concentrate were lower for cancer cells than for normal cells. Therefore, the additional use of a nanofiltration system to concentrate the obtained hydrolysate further increased the cytotoxicity of the product against cancer cells.

## Figures and Tables

**Table 1:** Chemical composition of the analyzed products.

Parameter	PJ	PJPH	cPJPH
Protein [%]	2.55 ± 0.11 ^c^	16.85 ± 0.12 ^b^	29.77 ± 0.23 ^a^
Ash [%]	0.97 ± 0.05 ^b^	23.02 ± 2.61 ^a^	24.34 ± 1.13 ^a^

PJ—fresh potato juice; PJPH—potato juice protein hydrolysate; cPJPH—concentrated potato juice protein hydrolysate. Mean values with different letters (^a–c^) in the rows are significantly different at α = 0.05.

**Table 2:** Mineral composition of the analyzed products.

Mineral	NRV [mg]	PJPH	cPJPH
K [mg/100 g]	2000	18823 ± 590	19623 ± 526
Mg [mg/100 g]	375	513 ± 10	664 ± 10
Na [mg/100 g]	N/A	161 ± 4	176 ± 4
Ca [mg/100 g]	800	160 ± 6	228 ± 5
Zn [mg/100 g]	10	6.02 ± 0.13	7.45 ± 0.15
Mn [mg/100 g]	2	6.18 ± 0.13	7.38 ± 0.12
Cu [mg/100 g]	1	1.19 ± 0.02	1.98 ± 0.07
Fe [mg/100 g]	14	0.44 ± 0.04	0.56 ± 0.08
Cd [μg/g]	-	10 ± 1	8.75 ± 0.14
Pb [μg/g]	-	4.31 ± 0.11	2.01 ± 0.13

PJPH—potato juice protein hydrolysate; cPJPH—concentrated potato juice protein hydrolysate; NRV—nutrient reference value; N/A—not applicable.

**Table 3:** The amino acid profile and amino acid score (AAS) for adults according to the standards reported by the FAO/WHO [52].

Amino Acid	FAO/WHO Standard [mg/g]	PJPH [g/16 g N]	AAS
Essential Amino Acids			
Histidine	16	1.92 ± 0.05	100
Isoleucine	30	2.44 ± 0.07	81.5
Leucine	61	2.35 ± 0.10	38.5
Lysine	48	2.28 ± 0.12	47.4
Methionine + Cystine	23	1.82 ± 0.21	79.0
Phenylalanine + Tyrosine	41	4.25 ± 0.33	100
Threonine	25	2.19 ± 0.08	87.7
Tryptophan	6.6	1.10 ± 0.08	100
Valine	40	4.44 ± 0.17	100
Dispensable Amino Acids			
Alanine	-	8.56 ± 0.29	-
Arginine	-	7.84 ± 0.31	-
Aspartic acid	-	15.06 ± 0.43	-
Glutamic acid	-	10.13 ± 0.50	-
Glycine	-	1.46 ± 0.22	-
Proline	-	1.69 ± 0.19	-
Serine	-	2.93 ± 0.13	-

**Table 4:** Antioxidant activity was expressed as Trolox equivalent antioxidant capacity (TEAC), and total phenolic compounds (TPC).

Parameter	PJPH	cPJPH
TEAC [mmol/g]	0.89 ± 0.05 ^b^	0.96 ± 0.03 ^a^
TPC [mg/g]	28.29 ± 1.88 ^b^	31.11 ± 2.16 ^a^

PJPH—potato juice protein hydrolysate; cPJPH—concentrated potato juice protein hydrolysate. Mean values with different letters (^a,b^) in the rows are significantly different at α = 0.05.

**Table 5:** Cytotoxic doses for stomach cancer cells (Hs746T line), colon cancer cells (HT-29 and Caco-2 lines), and colon normal cells (CCD 841 CoN line) [mg_dm_/mL].

Cell Line	IC_10_		IC_50_		IC_90_	
	PJPH	cPJPH	PJPH	cPJPH	PJPH	cPJPH
Hs 746T	4.43 ± 0.21 ^b^	1.80 ± 0.18 ^b^	6.30 ± 0.09 ^b^	2.95 ± 0.13 ^b^	8.96 ± 0.61 ^b^	4.84 ± 0.07 ^b^
Caco-2	2.58 ± 1.13 ^c^	0.55 ± 0.17 ^c^	5.26 ± 0.78 ^b,c^	1.62 ± 0.24 ^c^	11.35 ± 2.00 ^a^	4.88 ± 0.46 ^b^
HT-29	1.99 ± 0.19 ^c^	2.16 ± 0.31 ^b^	4.43 ± 0.21 ^c^	3.11 ± 0.16 ^b^	9.90 ± 0.61 ^a,b^	4.48 ± 0.20 ^b^
CCD 841 CoN	5.56 ± 0.30 ^a^	4.04 ± 0.09 ^a^	7.20 ± 0.10 ^a^	5.47 ± 0.01 ^a^	9.34 ± 0.39 ^a,b^	7.40 ± 0.16 ^a^

Mean values denoted by different letters (^a–c^) in columns differ statistically significantly (*p* < 0.05).

## Data Availability

The datasets generated during and/or analysed during the current study are available from the corresponding author on reasonable request.

## Sample Availability

Samples of PJPH and cPJPH are available from the corresponding author.
